# Case Report: Characterization of known (c.607G>C) and novel (c.416C>G) *ELANE* mutations in two Mexican families with congenital neutropenia

**DOI:** 10.3389/fimmu.2023.1194262

**Published:** 2023-09-18

**Authors:** María Enriqueta Núñez-Núñez, Juan Carlos Lona-Reyes, Brenda López-Barragán, Rosa Margarita Cruz-Osorio, Bricia Melissa Gutiérrez-Zepeda, Antonio Quintero-Ramos, Denisse Stephania Becerra-Loaiza

**Affiliations:** ^1^ Departamento de Alergia e Inmunología Clínica Pediátrica, Nuevo Hospital Civil de Guadalajara “Dr. Juan I. Menchaca”, Guadalajara, Mexico; ^2^ Departamento de Infectología, Nuevo Hospital Civil de Guadalajara “Dr. Juan I. Menchaca”, Guadalajara, Mexico; ^3^ Clínicas de Pediatría, Centro Universitario de Ciencias de la Salud, Universidad de Guadalajara, Guadalajara, Mexico; ^4^ Departamento de Pediatría, Nuevo Hospital Civil de Guadalajara “Dr. Juan I. Menchaca”, Guadalajara, Mexico; ^5^ Departamento de Hemato-Oncología Pediátrica, Nuevo Hospital Civil de Guadalajara “Dr. Juan I. Menchaca”, Guadalajara, Mexico; ^6^ Laboratorio de Inmunología, Departamento de Fisiología, Centro Universitario de Ciencias de la Salud, Universidad de Guadalajara, Guadalajara, Mexico; ^7^ Doctorado en Genética Humana, Departamento de Biología Molecular y Genómica, Centro Universitario de Ciencias de la Salud, Universidad de Guadalajara, Guadalajara, Mexico; ^8^ Unidad de Investigación Biomédica 02, Hospital de Especialidades, Centro Médico Nacional de Occidente, Instituto Mexicano del Seguro Social, Guadalajara, Mexico

**Keywords:** ELANE gene mutation, severe neutropenia, novel mutation, cyclic neutropenia (CyN), Mexican, c.416C>G, c.607G>C, case report

## Abstract

The most common causes of congenital neutropenia are mutations in the *ELANE* (Elastase, Neutrophil Expressed) gene (19p13.3), mostly in exon 5 and the distal portion of exon 4, which result in different clinical phenotypes of neutropenia. Here, we report two pathogenic mutations in *ELANE*, namely, c.607G>C (p.Gly203Arg) and a novel variant c.416C>G (p.Pro139Arg), found in two Mexican families ascertained via patients with congenital neutropenia who responded positively to the granulocyte colony-stimulating factor (G-CSF) treatment. These findings highlight the usefulness of identifying variants in patients with inborn errors of immunity for early clinical management and the need to rule out mosaicism in noncarrier parents with more than one case in the family.

## 1 Introduction

Congenital neutropenia (CN) encompasses a family of genetic diseases characterized by 1) low neutrophil counts (≤0.5–1.5 × 10^9^/L) and susceptibility to infection, 2) various organ dysfunctions, and 3) an extraordinarily high risk of leukemic transformation (1). The CN-specific manifestation of neutropenia can be a permanent condition in the patients or can occur in a cyclic/intermittent pattern where periods of normalization alternate with episodes of severe neutropenia (1). These diseases commonly result from mutations in the *ELANE* gene (19p13.3), which encodes for neutrophil elastase (NE) (EC 3.4.21.37), a pleiotropic enzyme pivotal for host innate defense, tissue remodeling, and local inflammatory responses, mediated by neutrophil and monocyte granules (2–4).

Heterozygous mutations in the *ELANE* gene cause autosomal dominant CN phenotypes exhibiting extremely high penetrance (5, 6). Mutations in exons 4 and 5 of *ELANE* may disrupt the disulfide bond domain in the C-terminus of the protease, which is essential for its correct intracellular localization. This disruption could lead to dysregulated vesicular sorting, trafficking, and reduced stability of mutant protein, manifesting as impaired neutrophil maturation and diverse CN phenotypes (2, 7). Identical *ELANE* mutations can cause severe congenital neutropenia (SCN) or cyclic/intermittent neutropenia (CyN) (8, 9) owing to neutrophil precursor arrest at the promyelocyte or myelocyte stage (4). Therefore, the clinical phenotype and natural history of patients should be assessed in the context of molecular findings.

Here, we report two *ELANE* mutations in three patients with CN from two families. One of them, a known mutation, formerly named c.607G>C (Gly203Arg), was observed in two sisters with different fathers who were diagnosed with SCN. To our knowledge, for the first time, a c.416C>G (p.Pro139Leu) mutation was associated with the clinical expression of CyN in a male child. We also reviewed literature on the identified mutations for this inborn error of immunity (IEI).

## 2 Case reports

### 2.1 Family 1

**Figure 1:**
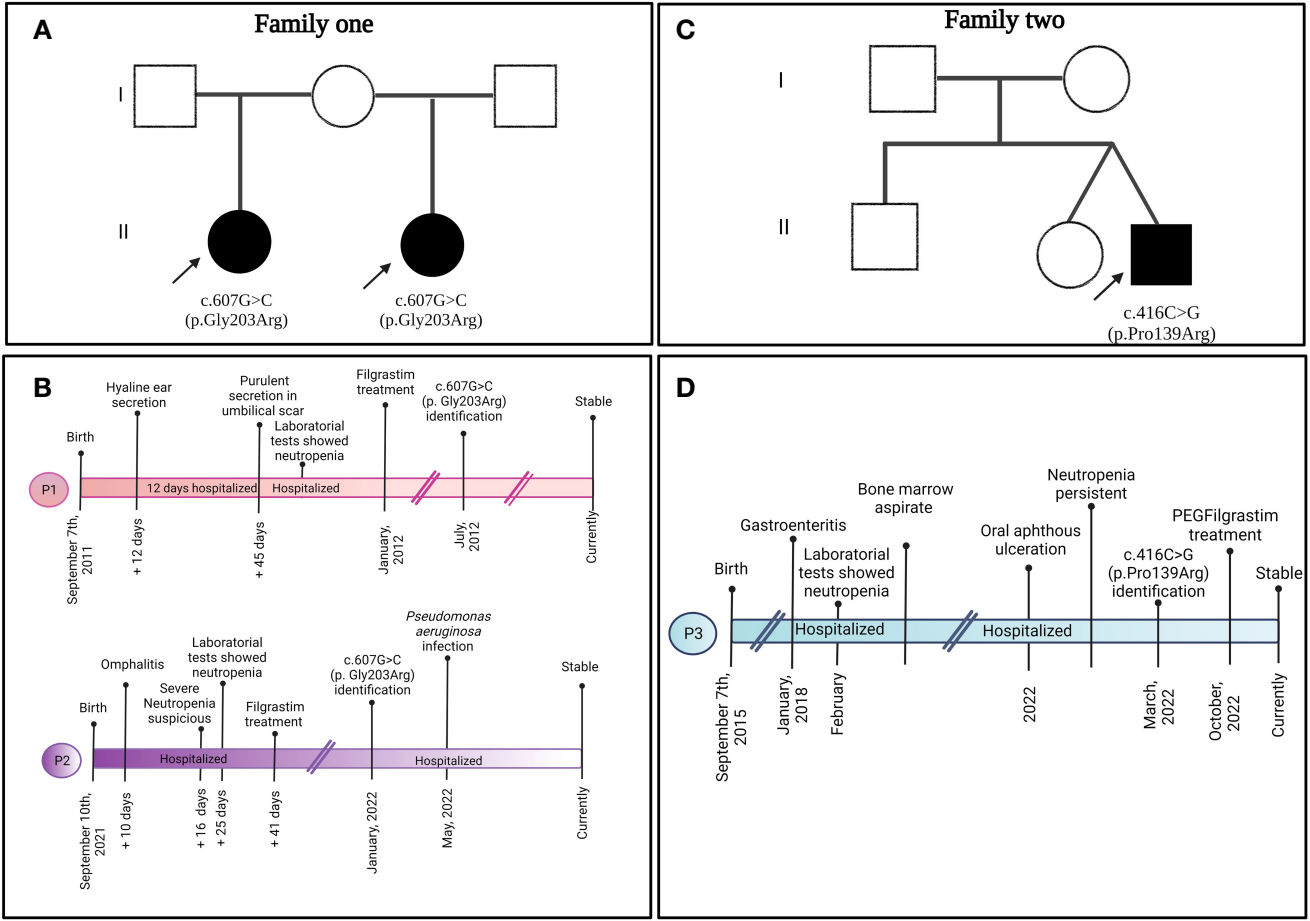
Familial pedigree of **(A)** family 1 and **(C)** family 2 depicting the clinical presentation timeline of **(B)** P1, P2, and **(D)** P3.

**Figure 2:**
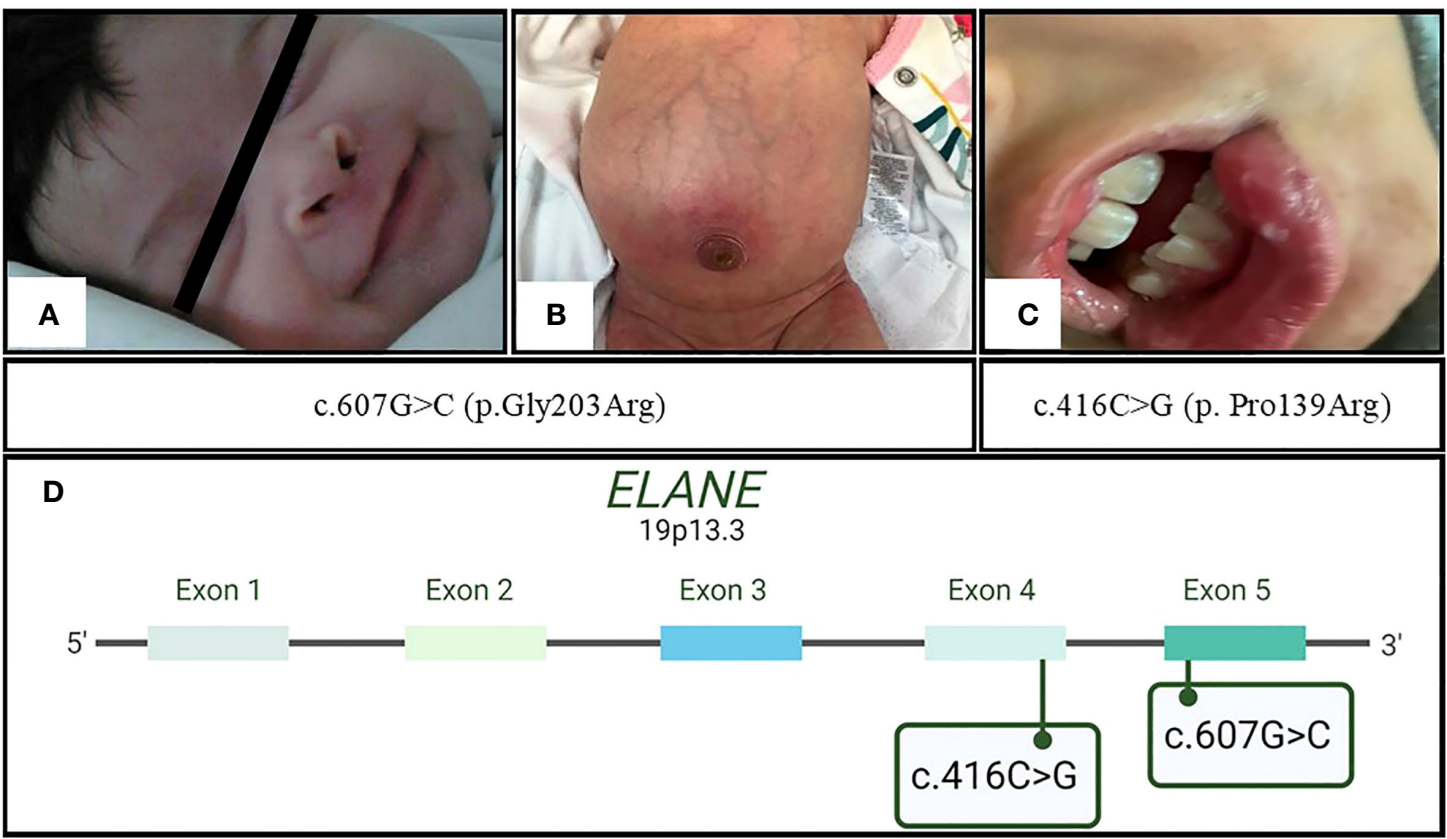
Initial clinical presentation of the patients. In family 1 with c.607G>C (p.Gly203Arg) mutation, **(A)** P1 with a necrotic lesion located in the right nostril and **(B)** P2 with omphalitis; in family 2 with novel mutation c.416C>G (p.Pro139Arg), **(C)** P3 with oral aphthous ulceration. **(D)** Ubication of the abovementioned *ELANE* mutations in exons 5 and 4, respectively.

Patient 1 (P1) (**Figures 1A, B**) was a female child born in September 2011 to a primiparous mother and an unrelated father from Jalisco, Mexico. P1 was hospitalized at 12 days of age due to irritability, fever, hyaline ear secretions, and suspected sepsis. At 45 days of age, she was hospitalized again with purulent secretions in the umbilical scar and infection in the soft tissues located in the right nostril (**Figure 2A**).

**Figure 3:**
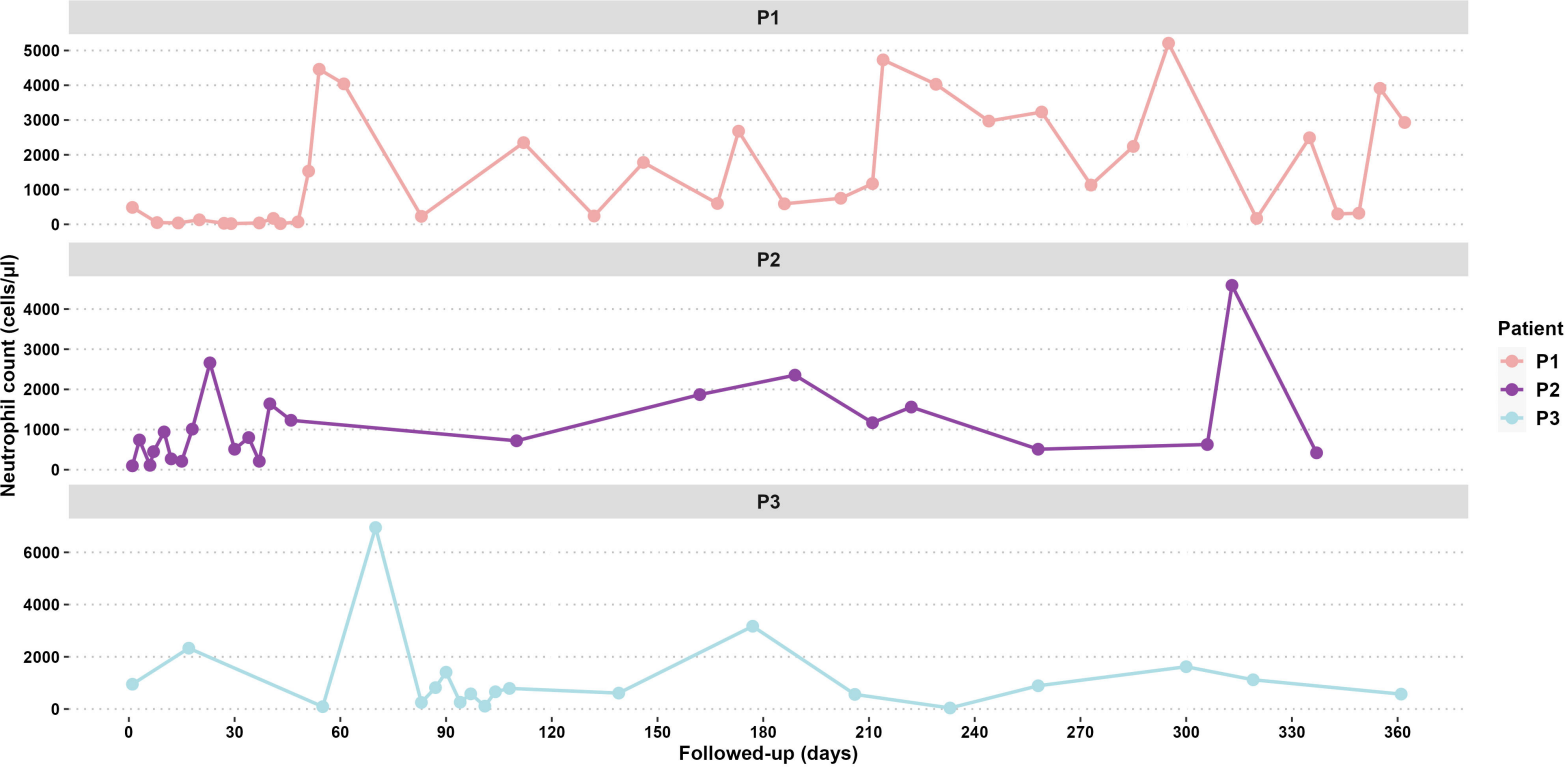
Follow-up neutrophil counts for 365 days of P1, P2, and P3.

Laboratory tests revealed neutropenia (50 cells/µL), anemia (8.1 g/dL), and monocytosis (3,930 cells/µL). The infection resolved after antimicrobial treatment, but neutropenia persisted with no improvement at 51 days of age. The bone marrow aspirate showed a 17% decrease in the number of monocytes and neutrophils. Immunoglobulins (Igs) and lymphocyte levels were normal: IgM = 132 mg/dL, IgG = 1,045 mg/dL, IgA = 89 mg/dL, IgE = 23.9 UI/dL; lymphocyte subsets CD3^+^ (3,495 cells/µL), CD4^+^ (2,522 cells/µL), CD8^+^ (922 cells/µL), and CD19^+^ (1,547 cells/µL). A second bone marrow aspirate revealed a decreased neutrophil count (7%), eosinophilia, and monocytosis but normal red blood cell counts. After eliminating other causes of neutropenia and considering the clinical course and persistent neutropenia (70 cells/µL, **Figure 3**), treatment with granulocyte colony-stimulating factor (G-CSF Filgrastim^®^; 30 µg/kg) was initiated at 3 months of age.

Patient 2 (P2) (**Figures 1A, B**) was the second female child born in September 2021 to the same mother, but from another non-consanguineous father from Jalisco, Mexico. P2 was hospitalized for omphalitis and neonatal sepsis (**Figure 2B**) at 16 days of age and treated with antibiotics; severe neutropenia was suspected (**Figure 3**). At 41 days of age, prophylactic antibiotic and Filgrastim^®^ treatments were initiated, but after 1 month, the dose was augmented from 30 µg/kg to 83 µg/kg per day. In May 2022, P2 was hospitalized again for a left inguinal abscess with erythema, high local temperature, and purulent secretions secondary to *Pseudomonas aeruginosa* infection.

Screening for gene mutations performed by Dr. Kaan Boztug (blood test in P1) or Invitae (buccal swab in P2) via sequencing [Sanger and Next-Generation Sequencing (NGS) panel, respectively] revealed that severe neutropenia in both patients was caused by the same pathogenic mutation, c.607G>C (p.Gly203Arg) (**Figure 2D**). The same tests on the parental samples revealed that neither parent carried the mutation (electropherograms were not available). At the time of the study, both patients followed their Filgrastim^®^ treatment (16 and 17 µg/kg per day for P1 and P2, respectively; average neutrophil counts: P1 = 2,300 cells/µL, P2 = 2,000 cells/µL), without clinical complications and good treatment response.

### 2.2 Family 2

Patient 3 (P3) (**Figures 1C, D**) was a male child born in September 2015 as the second twin of a second pregnancy of non-consanguineous parents (both from Jalisco). At 2 years 4 months of age, P3 was admitted to the emergency department because of severe dehydration secondary to gastroenteritis and was administered intravenous fluids. Laboratory results revealed bicytopenia (Hb = 9.6 g/dL and neutrophils = 100 cells/µL), whereas a bone marrow aspirate discarded a lymphoproliferative disorder. During the hospitalization, repeated neutrophil counts varied considerably (100 cells/µL, 4,850 cells/µL, 440 cells/µL, and 1,750 cells/µL); after 7 days, P3 was discharged.

Four years later, P3 developed intermittent fever accompanied by monthly oral aphthous ulceration and was rehospitalized (**Figure 2C**). On this occasion, the hematological parameters were normal, except for the neutrophil count, which ranged from 60 to 610 cells/µL with two peaks of 6,950 cells/µL and 1,410 cells/µL. Neutropenia persisted from November 2021 until August 2022, as diagnosed in serial blood counts performed twice per week during April and May (**Figure 3**), which also showed normal lymphocyte subpopulation counts and Ig levels. To corroborate the diagnosis of CyN, sequence analysis and deletion/duplication testing of a panel of 574 genes were kindly provided by Invitae; unfortunately, the electropherograms were not available.

P3 was heterozygous for variants of uncertain significance (VUSs) in genes for ATP-binding cassette transporters G5 [*ABCG8* (c.255G>T, p.Glu85Asp)], Ankyrin repeat and zinc peptidyl TRNA hydrolase [*ANKZF1* (c.1053A>C, p.Glu351Asp)], Centrosomal protein 164 [*CEP164* (c.1152A>G, silent)], Cystic fibrosis transmembrane conductance regulator [*CFTR* (c.335A>G, p.Asp112Gly)], Forkhead box N1 [*FOXN1* (c.724C>T, p.Pro242Ser)], and SKI2 subunit of superkiller complex [*SKIV2L* (c.2165G>A, p.Arg722Gln)]. Furthermore, a novel pathogenic variant, c.416C>G (p.Pro139Arg) (**Figure 2D**), in *ELANE* was found. The same tests on the parental samples revealed that neither parent carried these variants. In September 2022, Filgrastim^®^ treatment (5 µg/kg per day) was initiated; however, since October 2022, P3 followed subcutaneous PEGFilgrastim^®^ treatment (1 mg) every 21 days. Currently, P3 presents an average neutrophil count of 1,350 cells/µL, without clinical manifestations and good treatment response.

## 3 Discussion

In this report, P1 and P2 exhibited the same pathogenic mutation, namely, autosomal dominant c.607G>C (p.Gly203Arg) (rs201139487) located in exon 5 of *ELANE*, which could account for the nonfamilial SCN of the patients. This mutation was reported for the first time in a Korean girl with SCN (10), with an initial clinical presentation of omphalitis, like that observed in P1 and P2. However, in our case, the clinical diagnosis of P1 and P2 was done earlier (at 4 months of age) than in the case of the Korean girl (at 9 months of age).

The existence of genetically distinct populations of cells in a particular organism, called mosaicism, is an important cause of genetic disease and can appear as *de novo* DNA mutations (11). Accordingly, Shigemura et al. (12) reported a c.607G>C *ELANE* mutation in an asymptomatic Japanese mother, who exhibited mosaicism, whose daughter had SCN; however, this report did not provide a clinical description of the patient. Similarly, paternal mosaicism for other *ELANE* mutations causing SCN has been reported (13–15). Accordingly, in the present study, germline mosaicism was suspected in the unaffected mother of both patients carrying the c.607G>C mutation, which is relevant considering only a few families have presented mosaicism of mutation in the *ELANE* gene (13).

Sporadic CyN is caused by *de novo* mutations in the *ELANE* gene (16). Clinically, patients exhibit fever, infections, and neutropenia, with blood neutrophil counts fluctuating approximately every 14–35 days and profound neutropenia for 3–10 days (17, 18). The clinical evaluation of these events in CyN can be challenging, as observed with P3, whose neutropenia showed irregular fluctuations, and responded to therapy (19). In P3, the neutrophil counts represented the intermittent neutropenia (CyN) pattern with profound neutropenia alternating with peaks of elevated neutrophil counts in a period of 6 weeks; this sufficiently confirmed the diagnosis based on the pediatric hematology protocol of Hospital Civil Nuevo “Dr Juan I Menchaca” and according to the literature (20–22).

The novel mutation in the distal portion of exon 4, c.416C>G (p.Pro139Arg), found in P3 resembles the c.416C>T (p.Pro139Leu) (rs137854448) (23) pathogenic single-nucleotide variant (SNV) in the same codon that causes both CyN and SCN (24–26). The major concern in CN mortality is the progression and development of leukemia (27), which is related to certain germline mutations (26). In this respect, the previously reported SNV c.416C>T (p.Pro139Leu) (rs137854448) (24) has been associated with a good prognosis without an increased risk of leukemia development (28). On the other hand, the mechanism by which defects in NE lead to leukemia is unknown and there is still a lack of hallmark predictors (29). G-CSF treatment is known to increase the risk of leukemia development by 22% after 15 years of treatment, and a higher dose or diminished response augments these probabilities (30, 31). Previously, Freedman et al. (32) disregarded any relationship between age, sex, dosage or duration of treatment, and malignant transformation; thus, the associations are still unclear. However, during treatment, marrow bone cells acquire somatic mutations related to myelodysplastic syndromes/acute myeloid leukemia (MDS/AML) (33–35). Therefore, we suggest that some germline mutations can interact with acquired somatic mutations and moderate the clinical presentation.

Invitae catalogs the missense mutation c.416C>G (p.Pro139Arg) (SCV003915652) as pathogenic, derived from algorithms that predict the disruptive effect of Pro/Arg changes on the protein structure and function. However, these are limiting *in silico* speculative conjunctions. Follow-up of P3 might demonstrate whether this novel mutation is related to leukemia. The clinical expression of CN (SCN or CyN), depending on the mutation, is homogeneous or variable, suggesting different pathogenic mechanisms (36). Previous *in silico* analyses showed a potentially high risk of altered protein structure and function for c.607G>C (rs201139487) and c.416C>G (rs137854448) (37) located in the same codon of SNV reported here for the first time. Additionally, Shinwari et al. (37) reported that these SNVs were related to SCN and CyN (24, 25).

Moreover, the synergistic effect of concurrent mutations (38–40) explains the distinct pathomechanisms of phenotype determination aside from modifier genes (41). Significantly, the concomitant mutations in P3 affected the interacting molecules, namely, *ABCG8*, *ANKZF1*, *CEP164*, *CFTR*, *FOXN1*, *SKIV2L*, and *ELANE* (42, 43). According to Invitae’s report, none of these has been found in CyN or SCN; in the absence of sufficient information to determine their role in CyN, they have been classified as tolerated variants by bioinformatic predictions (SIFT, Polyphen-2, and Align-GVGD). Moreover, the present case of sporadic CyN with multiple mutations could have resulted from a higher germline mutation rate in older males than those in females (44). In addition, *ELANE* is prone to somatic mutagenesis (40). Thus, our report of the concurrence of these VUSs with *ELANE*, interacting and co-expressing with many genes (42, 43), enhances the data of new mutations in patients with IEI and may enable a more effective classification of clinical features and complications.

When there is insufficient evidence linking the variant to disease and a VUS is reported, it creates a problem for the clinician in interpretation and management of the patient (45). However, reporting the VUS in different clinical presentations could support the reclassification and genetic testing strategies, thus helping physicians understand how to incorporate these results into clinical care (46, 47). Therefore, it is essential to review the literature on common clinical features and phenotypic variations among affected individuals (48). Our findings highlight the usefulness of identifying variants in patients with IEI, such as CN, for early clinical management and the need to rule out mosaicism in apparently noncarrier parents with more than one case in the family, although for that, this report is speculative. Furthermore, we report a novel c.416C>G (p.Pro139Arg) mutation in a male child diagnosed with CyN. This report enriches the clinical mutation database and can also help further and improve management of patients worldwide with similar clinical manifestations.

## Data availability statement

The datasets presented in this study can be found in online repositories. The names of the repository/repositories and accession number(s) can be found below: SCV003915652 (ClinVar).

## Ethics statement

The studies involving humans were approved by Comité de ética e investigación Hospital Civil de Guadalajara Dr. Juan I. Menchaca. The studies were conducted in accordance with the local legislation and institutional requirements. Written informed consent for participation in this study was provided by the participants’ legal guardians/next of kin. Written informed consent was obtained from the individual(s) for the publication of any potentially identifiable images or data included in this article. Written informed consent was obtained from the participant/patient(s) for the publication of this case report.

## Author contributions

JL-R, BL-B, and RC-O contributed to the conception of the case report and acquired data. MN-N, DB-L, BG-Z, and AQ-R acquired, analyzed, and interpreted the patient data. DB-L and MN-N drafted the manuscript. All authors contributed to the article and approved the submitted version.
